# Impact of iodine supplementation during preconception, pregnancy and lactation on maternal thyroid homeostasis and offspring psychomotor development: protocol of the IodineMinho prospective study

**DOI:** 10.1186/s12884-020-03376-y

**Published:** 2020-11-13

**Authors:** Maria Lopes-Pereira, Susana Roque, Patrício Costa, Anna Quialheiro, Nadine Correia Santos, Ana Goios, Laura Vilarinho, Margarida Correia-Neves, Joana Almeida Palha

**Affiliations:** 1 Life and Health Sciences Research Institute (ICVS), School of Medicine, University of Minho, Campus Gualtar, 4710-057 Braga, Portugal; 2 ICVS/3B’s, PT Government Associate Laboratory, Braga/Guimaraes, Portugal; 3 Hospital de Braga, Braga, Portugal; 4 Newborn Screening, Metabolism & Genetics Unit, National Institute of Health Dr Ricardo Jorge, Porto, Portugal; 5 Clinical Academic Center, Braga, Portugal

**Keywords:** Iodine, Iodine deficiency, Iodine supplementation, Pregnancy, Nutrition, Psychomotor development, Public health intervention, Maternal health, Child health

## Abstract

**Background:**

Iodine deficiency is the most common cause of preventable brain harm and cognitive impairment in children. Portuguese women of childbearing age, pregnant women and their progeny were shown to have inadequate iodine intake. Consequently, the Portuguese Health Authorities have recommended a daily supplementation with 150–200 µg iodine in preconception, pregnancy, and lactation. The IodineMinho study intends to evaluate whether (i) this recommendation impacted on the prevalence of iodine deficiency in pregnant women from the Minho region of Portugal, (ii) the time of initiation of iodine supplementation (if any) influences the serum levels of thyroid hormones at several intervals during pregnancy and (iii) there are serum thyroid-hormone parameters in the 1st trimester of pregnancy that predict psychomotor development of the child at 18 months of age.

**Methods:**

Most Portuguese women are followed throughout pregnancy in community Family Health Units, where family physicians may choose to follow the National recommendation or other, concerning iodine sufficiency. This study will recruit women (N = 304) who intend to become pregnant or are already pregnant from 10 representative Units. Physician’s approach and prescriptions, sociodemographic, nutrition and clinical information will be obtained at baseline and throughout pregnancy. To evaluate endocrine function, blood and urine samples will be collected at recruitment, once in each trimester of pregnancy, at delivery and 3 months after delivery. Breastmilk samples will be collected for iodine and energy content analysis. Children will be evaluated for psychomotor development at 18 months. Maternal thyroid volume will be evaluated by ultrasound scan at baseline, in the 3rd trimester and at 3 months after delivery.

**Discussion:**

Iodine deficiency early during development precludes children from achieving full intellectual capabilities. This protocol describes a study that is innovative and unique in its detailed and comprehensive evaluation of maternal and child endocrine and psychomotor parameters. By evaluating the effectiveness of the iodine supplementation recommendation, it will contribute to the public health systems’ efforts to provide excellence in maternal and infant care.

**Trial registration:**

ClinicalTrials.gov, NCT04288531. Registered 28 February 2020-Retrospectively registered.

## Background

Iodine is needed to synthesize thyroid hormones, which regulate metabolism and early fetal brain development. Iodine deficiency is the most frequent preventable cause of profound intellectual disability in the world. While cretinism, the most severe consequence of iodine deficiency, has been eradicated due to disseminated strategies for iodine supplementation [1], iodine deficiency remains a global health problem [2]. Insufficient iodine intake is particularly relevant in conditions of increased iodine need, such as pregnancy. It is well known that the 1st trimester of pregnancy is a critical window for the irreversible impact of thyroid hormones on brain development [3]. Insufficient iodine intake is responsible for the failure of many individuals to achieve their full intellectual capabilities, which has great implications for any given generation.

At present, pregnant women are moderately iodine deficient worldwide, including in several European countries [1–3]. In the Minho region of Portugal, we showed in 2009 that the median urinary iodine concentration (UIC) was below the desired values in women and their progeny (in 61% of women before pregnancy, 76–91% of pregnant women, 66–92% of women after pregnancy, 62% of newborns, and 52% of infants). Of notice, UIC was lower in infants fed on breastmilk than those fed on formula milk [4]. We also observed that free thyroxine (T4) levels in the 1st trimester were lower than expected for this period of pregnancy [5] and that this parameter predicted the psychomotor development of the progeny [6]. In a subsequent study on 3631 pregnant women in Portugal, an adequate UIC (> 150 ug/L) was observed in only 17% of those in mainland Portugal, 8.2% of those in Madeira islands and 2.3% of those in the Azores islands (where 50% of pregnant women had UIC < 50 ug/L) [7]. These results were surprising for a country with such a large coastal area, and prompted the Portuguese Health Authorities to issue, in August 2013, a recommendation for iodine supplementation (150–200 µg/day) prior to and throughout pregnancy, and until the end of lactation [8].

**Fig. 1:**
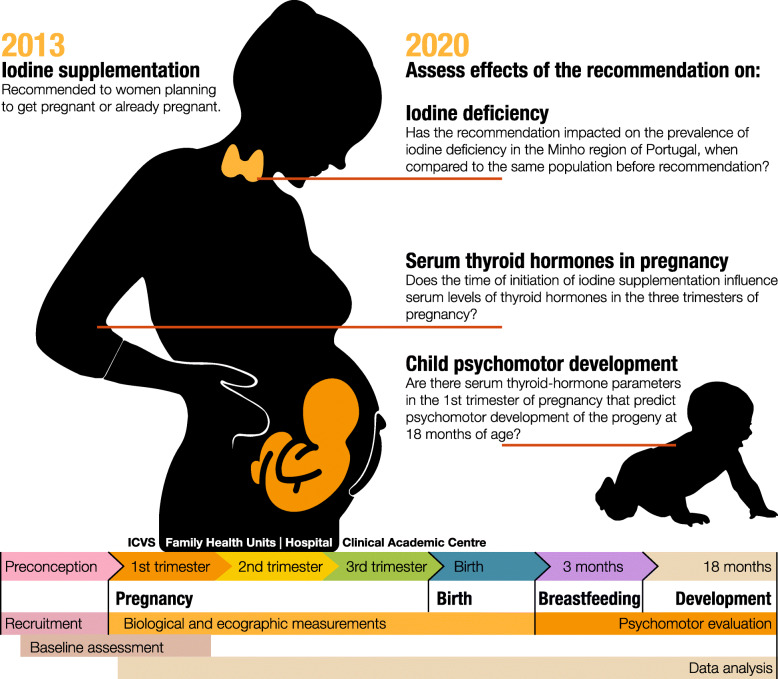
Study overview and aims (This is an original image by the authors)

Several studies have reported that iodine supplementation in pregnancy helps in normalizing maternal thyroid function and in the development of the newborn. In contrast, others found no such evidence [9–11]. Most of these studies had methodological limitations, such as the timing of iodine supplementation, the number of assessment periods, the number of mother/child pairs or the number of variables considered in the analysis. Consistent with this, the latest Cochrane review on “Iodine supplementation for women during the preconception, pregnancy and postpartum period” concluded that we lack sufficient evidence to conclude on the benefits and harms of iodine supplementation, and that further well-designed studies are necessary [12]. This provides an opportunity for the present study and underscores its relevance (Fig. 1).

## Methods/design

### Aims

Primary aims:

- Has the new policy of recommending iodine supplementation in preconception, throughout pregnancy and during lactation impacted the prevalence of iodine deficiency in pregnant women from the Minho region of Portugal when compared to the same population before the recommendation [6]?
- Does the time of initiation of iodine supplementation (if any) influence the serum levels of thyroid hormones at several intervals during pregnancy?
- Are there serum thyroid-hormone parameters in the 1st trimester of pregnancy that predict psychomotor development of the child at 18 months of age?

Secondary aims:

The comprehensive analysis of biochemical parameters in the various trimesters of pregnancy and during lactation will also allow to address the following questions:

- What are the standard reference ranges for thyroid hormone parameters throughout pregnancy (not yet established for the Portuguese population)?
- Are there thyroid function parameters (e.g., the presence of anti-thyroid antibodies) that predict the response to iodine supplementation?
- Is the quality of breastmilk related to thyroid hormone parameters in a way that could suggest new intervention strategies for real-time milk fortification during lactation?
- Are the fetal heart rate and thyroid volume at age 3 months influenced by maternal iodine status?

### Design

An observational prospective cohort study of maternal/child pairs, from preconception, pregnancy and until 18 months of age of the child.

The study involves the following time points for assessment (Table 1):

- M0-Baseline evaluation. Women before pregnancy (for planned pregnancies), at the beginning of pregnancy (for unplanned pregnancies) and women of childbearing age and not intending to become pregnant.
- M1-M2-M3-Women in each trimester of pregnancy.
- M3-Fetus.
- M4- Women and newborn. Delivery.
- M5-Women and progeny three months after delivery.
- M6-Women and progeny eighteen months after delivery

**Table 1:** Longitudinal assessment timepoints: when, who, where.

Moment		Evaluation	Filled in by:	Local
M0	Recruitment (preconception or 1st trimester)	Informed consent	Participant	Family health centers
		Sociodemographic evaluation	Participant	
		Clinical evaluation	Clinician	
		Food frequency questionnaire	Participant	
		3 days food record	Participant	Home
		Blood and urine collection	Clinical Analysis Laboratory	
		Thyroid ultrasound	Clinician	2CA
M1	1^st^ trimester of pregnancy	Clinical evaluation	Clinician	Family health centers
		Blood and urine collection	Clinical Analysis Laboratory	
M2	2^nd^ trimester of pregnancy	Clinical evaluation	Clinician	Family health centers
		Blood and urine collection	Clinical Analysis Laboratory	
M3	3^rd^ trimester of pregnancy	Clinical evaluation	Clinician	Family health centers
		Blood and urine collection	Clinical Analysis Laboratory	
		Thyroid ultrasound	Clinician	2CA
		Fetal cardiotocography	Clinician/Nurse	Hospital Braga
M4	Birth	Clinical evaluation	Clinician	Hospital Braga
		Blood and urine collection - pregnant	Nurse	
		Urine collection– Newborn		
M5	3 months after birth	Breast milk collection	Nurse	2CA
		Blood and urine collection – puerperal women		
		Urine collection – newborn		
		Thyroid ultrasound - puerperal women	Clinician	
		Thyroid ultrasound - child		
M6	18 months after birth	Child psychomotor evaluation	Researcher	2CA
		Blood and urine collection –women	Nurse	
		Urine collection – child		

### Intervention

The National Health Authorities have issued a recommendation for iodine supplementation (150 to 200 µg potassium iodide/day, in tablet form) during preconception, pregnancy, and lactation [8]. The recommendation is not mandatory and does not provide detailed instructions on specific timings to initiate and terminate this supplementation. The practice of doctors/Family Health Units with respect to iodine supplementation will be registered without any interference/guidance from the study researchers. The compliance with iodine supplementation intake will be monitored by the Family Health Units (doctors and nurses).

### Setting and participants

Women will be recruited in 10 Family Health Units (recruitment has started successfully in a feasibility study in 2 Units) and followed during pregnancy. Family doctors will present the study and collect informed consent at the time of the first visit. During pregnancy, clinical information and biological fluid collection will coincide with the routine follow-up visits. Participants will be invited to the Clinical Academic Center-Braga (2CA-Braga) at recruitment and in the third trimester of pregnancy for a thyroid ultrasound, at 3 months after delivery for thyroid ultrasound (mother and child), breast milk, blood and urine collection, and at 18 months for blood (mother) and urine (mother and child) collection and for psychomotor evaluation of the child. Immediately after delivery, in the Braga Hospital, urine (mother and child) and blood (mother) will be collected.

Family doctors may not recruit all potential participants from the study population. To control for selection bias, aggregated information will be collected at each recruitment site on age, number of children, education, and reason for not recruitment (not invited/declined participation) of potential participants.

### Inclusion criteria

Women in preconception or 1st trimester of pregnancy, confirmed according to amenorrhea, recruited in the Family Health Units selected for this study, who intend to deliver in the Public Braga Hospital and provide informed consent.

### Exclusion criteria

Women transferred to or from outside the study’s Family Health Units during pregnancy, women who do not intend to deliver at the Public Braga Hospital, women already taking iodine supplementation at the time of enrollment and pregnant women with gestational age further than 13 weeks of gestation.

### Study parameters

SOCIO-ECONOMIC status (Graffar Scale) [13, 14] and NUTRITIONAL status (3-day food record [15] and food frequency questionnaire [16, 17] will be assessed by questionnaires administered at M0.

CLINICAL INFORMATION, such as medication, clinical history and any complications (e.g., spontaneous abortion, preterm delivery, placenta abruption, hypertension and preeclampsia, gestational diabetes, hydramnios, low birth weight, fetal death) will be registered at the trimestral (M0-M3) routine visits to the doctor.

BIOCHEMICAL parameters for women [urinary and breastmilk (only in M5) iodine, and serum anti-peroxidase and anti-thyroglobulin antibodies, total and free T4 and triiodothyronine (T3), thyroxine binding globulin (TBG), thyroid stimulating hormone (TSH), thyroglobulin (TG) (M1, M4 and M5) and human chorionic gonadotropin (hCG) (only in M1)] will be assessed at M0-M6 while those for newborns [TSH and T4 (from the neonatal screening program, provided the National Institute of Health) and urine iodine] will be sampled at M4 and M5. Biochemical endocrine measurements will be done at certified clinical laboratories. Urine and breast milk iodine will be measured using the Sandell-Kolthoff reaction method. [18] All the hormones and antibodies will be measured by quantitative chemiluminescent immunoassays. Anti-peroxidase and anti-thyroglobulin antibodies, total and free T4 and T3, TSH and TG will be quantified using the Siemens Atellica IM system; TBG and hCG will be measured in the Siemens Immulite 2000 Xpi system.

MATERNAL AND CHILD THYROID VOLUME will be evaluated by ultrasound at M0, M3 and M5, for women; M5 for the child, in the 2CA-Braga.

FETAL HEART RATE: Fetal cardiotocography at M3 in the 2CA-Braga.

BREASTMILK will be assessed for energy content (fat, protein and carbohydrates) using a commercial milk analyzer (at M5) and for iodine.

PSYCHOMOTOR DEVELOPMENT of the progeny at 18 months of age will be assessed using the Bayley Scales of Infant and Toddler Development (III) [19]. This instrument is one of the most reliable and widely used to evaluate children development at 1–42 months. Our group and others have previously used licensed Bayley Scales to address infants’ psychomotor performance in the context of maternal hypothyroxinemia [6, 20, 21]. It includes three subscores: cognitive (information processing, information processing speed, problem-solving, play skills and numbers concepts); language communication (expressive, which is the ability to communicate, and receptive, which includes the abilities to hear, understand and respond) and motor development (fine and gross motor abilities, including movement quality, sensory and perceptual-motor integration and basic locomotion milestones). Scores are scaled from 40 to 160, and a child is considered to have a developmental delay when they score lower than 85 on one of the subscores. Scores will be corrected for premature babies [22].

### Sample size calculation

Considering the primary questions of the study, the sample sizes were calculated as follows:

- Regarding prevalence. For a statistical power of 99%, a type I error of 1%, a previous proportion of 88% iodine deficiency [4] and assuming a 70% decrease in the number of women with iodine deficiency, the required sample size is 122 participants (pregnant women). A comparison group of 122 women who do not plan to become pregnant will be enrolled from the same health care units to control for other environmental factors that may influence iodine intake and the prevalence of iodine deficiency.
- Regarding pregnancy thyroid hormone levels. For a statistical power of 99%, a type I error of 1%, a previous proportion of 17% women with the expected increase in total T4 in the 1st trimester of pregnancy [5], and assuming a 2-fold increase in this number, the required sample size is 304 participants.
- Regarding the prediction of psychomotor development. For a statistical power of 99%, a type I error of 1%, an Odds Ratio of 2 and Pr(Y = 1|X = 1) H0 = 0.25 (probability of the parameter to be studied being below percentile 0.25), the required sample size is 262 participants.

Given the numerous time points that will be assessed (see below), a conservative attrition rate of 50% is being used to calculate an overall sample size of 608. The pilot feasibility study revealed a 5% drop-out (1/22) in the 2nd assessment.

### Data analysis

Continuous variables will be checked for normality using the Shapiro-Wilk normality test. Differences between groups (no iodine supplementation, iodine supplementation starting in preconception, or any of the trimesters of pregnancy) will be analyzed by Independent t-test or Mann-Whitney test, and ANOVA or Kruskal-Wallis test, depending on whether normally or not normally distributed. Categorical variables will be compared among groups using the Chi-square test.

In this longitudinal design, the trajectories will be studied using mixed and latent growth models. This strategy combines within-subject and between-subject factors and will determine the format of the curve and which variables shape its longitudinal trajectory.[23].

Unsupervised models will be used to create groups concerning psychomotor development. Cluster analysis will be applied to the standardized variables. By applying a hierarchical model approach using Ward’s linkage and Euclidean distances, the number of clusters will be decided based on the “elbow” method. Then, cluster centroids will be obtained for the optimal cluster solution and used in the k-means clustering method.[24].

The relationship between variables will be addressed using structural equation modeling, path analysis and multiple linear or logistic regression models. These procedures will identify the main predictors (and relevant interactions between predictors, as well as their moderation effects) of the children’s psychomotor development.[25].

The receiver operator characteristic analysis curves and Youden index will be used to determine the optimal cut-off value of the thyroid hormone parameters that can be used to predict psychomotor development impairment.

There will be no imputation for missing values. Depending on the research question and respective statistical method, only the full valid information will be considered.

A sensitivity analysis will be conducted to analyze whether attrition might have biased the results. Two-tailed *p* < 0.05 will be considered statistically significant. Statistical analyses will be performed using the R software (Lavaan package) and IBM SPSS Statistics Amos v.26.

## Discussion

It should be stated that the study poses no risks for women or their offspring.

Given the longitudinal design and the number of time-points a contingency plan was prepared to address the following concerns:

- Insufficient number of participants: Although the target sample size already includes a dropout rate of 50%, the study can be extended until the expected number is reached. However, from our experience, attrition is below 20% in studies followed at the 2CA-Braga. In addition, pregnant women are highly motivated in studies that provide information on the health of their children.
- Delays in follow-up: This is not anticipated since consultations/evaluations will be scheduled within a 2-week interval from the scheduling contact.
-  Participants forgetting to present for collection of blood or urine: Team members will follow all steps and will contact participants if appointments are missed.

Main potential biases:

- Selection bias: It is possible that, depending on the acceptance rate, those who enroll will differ from those who do not accept or are not invited to participate. Recruitment sites will provide general information on women who are not invited or do not agree to participate.
- Detection bias: Outcome measures will be related to the iodine supplementation and iodine status. Researchers evaluating outcome measures will be blind to when iodine supplementation was initiated.

In summary, this study was designed to evaluate the effectiveness of a recommendation for iodine supplementation before and during pregnancy and lactation, in correcting iodine nutritional levels and to understand the adequacy of the timing of such supplementation. It will also provide a unique detailed and comprehensive evaluation of maternal and child endocrine and psychomotor parameters. Overall, it will contribute to the public health systems’ efforts to provide excellence in maternal and infant care.

## Data Availability

The datasets used and/or analyzed during the current study will be available from the corresponding author on reasonable request.

## Abbreviations

2CA-Braga: Clinical Academic Center-Braga
T4: Thyroxine
TSH: Thyroid stimulating hormone
UIC: Urinary iodine concentration
